# Equity analysis of older adult resource allocation in China

**DOI:** 10.3389/fpubh.2024.1411054

**Published:** 2024-07-12

**Authors:** Qianqian Yu, Tiantian Zhang, Luyi Jiang, Yun Jia, Yuxing Dong, Li Luo

**Affiliations:** ^1^Shanghai Institute of Infectious Disease and Biosecurity, Fudan University, Shanghai, China; ^2^School of Management, Shandong Second Medical University, Shandong, China; ^3^School of Public Health, Fudan University, Shanghai, China; ^4^Shanghai Health Development Research Center (Shanghai Medical Information Center), Shanghai, China; ^5^Department of Health Management, PICC Health Insurance Company Limited, Beijing, China

**Keywords:** older adult resource allocation, equity analysis, agglomeration analysis, Gini coefficient, Lorenz curve

## Abstract

**Objective:**

To evaluate the current status and equity of older adult resource allocation in the 31 provinces (autonomous regions and municipalities) of mainland China, and to offer recommendations for the optimization of these allocations.

**Methods:**

Four key indicators, namely, the number of older adult institutions, employees, professionals, and beds in mainland China in the year 2020, were used and analyzed using various methods and tools, including agglomeration analysis, the Gini coefficient, and the Lorenz Curve. These methods were applied to evaluate the equity of older adult resource allocation across the different provinces (autonomous regions and municipalities) and regions of China, using two dimensions, namely, the geographical area and the older adult population.

**Results:**

Overall, the number of older adult resource allocations was found to be increasing in China, while the number of employees with educational levels of junior college or above was relatively low and the population structure was aging. In terms of the equity of older adult resource allocation, the results showed that this was good according to the dimensions of the older adult population but was on the low side based on the dimension of geographical area, and the Gini coefficient of the western region, in particular, was in an alarming state. Different provinces (autonomous regions and municipalities) were found to have an uneven allocation of resources for older adults, with large differences, with some areas having a serious under-allocation of resources, while others showed resource over-allocation.

**Conclusion:**

While China’s allocation of older adult resources is relatively equitable, there is nevertheless a need to take into account recent changes in the older adult population and strengthen the construction of a reasonably structured, high-quality team of professionals and technicians, as well as consider factors such as geographical area and the older adult population, and rationally allocate older adult resources in the eastern, middle, and western regions, to achieve a balanced allocation in terms of equity and efficiency and enhance social capital, to better satisfy the demands for older adult services in older adults at multiple levels.

## 1 Introduction

Since 2022, China’s demographic situation has undergone significant changes, with the total population entering a negative growth phase accompanied by the aging of the population (1). According to the China Statistical Yearbook 2023, at the end of 2022, there were 209.78 million older adults over the age of 65 years old in China, accounting for 14.9% of the total population. The Chinese Committee on Aging predicts that by 2050, the number of older adults in China will reach 487 million, representing close to 35% of the overall population indicating that China will have become a heavily aging society (2). China has the world’s largest population of older adults and the rapid pace of population aging, accompanied by reductions in family sizes, childlessness, uneven regional development, and the effect of “aging before getting rich” will lead to a series of adverse effects. First, as the older adult population base rises, the social dependency ratio increases, and the burden to the state, society, and the family gradually increases. Secondly, the growing demand for life care, rehabilitation care, medical care, and psychological comfort by older adults, as well as the sharp increase in the demand for social older adult services and medical and health services, and the substantial increase in the cost of medical and health services for the State and individuals, have increased the pressure on the income and expenditure of social older adult insurance and medical insurance funds, and the supply of resources for medical and health services and payment of costs have come under enormous pressure. Third, the aging of the population affects the ability of the healthcare system to raise funds, leading to a lack of human resources in the labor market and slower economic growth. Demographic aging is the key point of the current population problem in China, and an active response to the situation is necessary (3).

Facing the increasingly serious problem of aging in China, the government has introduced a series of policies. For example, in 2019, the Central Committee of the Communist Party of China and State Council issued *the National Medium-and Long-Term Plan for Actively Responding to Population Aging*, which is a strategic, comprehensive, and guiding document that proposes to improve the senior-care service system at multiple levels based on the home as a foundation, the community as a support, the full development of institutions, and the organic combination of medical care and nourishment, setting developmental goals for different periods (4). In 2021, The Central Committee of the Communist Party of China and State Council issued *The Opinions on Strengthening the Work of the Old Adult in the New Era*, emphasizing the integration of the concepts of active aging and healthy aging into the whole process of economic and social development, the improvement of the health support system for the older adult, and the enhancement of the level of health services and management for the older adult (5). In 2021, *The 14th Five-Year Plan for the Development of the National Aging Career and Older Adult Service System, In 2022,The 14th Five-Year Plan for Healthy Aging*, and other documents were issued to refine the objectives of the active response to the aging of the population, and to propose the establishment of an older adult service system that is compatible with population aging, to continuously expand the inclusive older adult service system, and to establish an older adult service system that is compatible with the overall aging of the population. These documents also propose the establishment of an older adult service system that is in line with the aging of the population, with the continuous expansion of inclusive older adult service resources (6, 7). The successive issuing of these policies has provided strategic guidance for localities to promote the construction of active aging, and different regions, based on the planning objectives, have actively explored the construction of the older adult care system and the allocation of older adult care resources as well as the supply of older adult care services and other dimensions, with significant results. At present, China’s older adult resources have been continuously improved in terms of both quantity and quality, thus improving the level of health and meeting the needs of older adults in the population.

To cope with the increasing demand of the older adult population, the Chinese government has continuously promoted the optimal allocation of older adult resources (8). A series of studies have also been conducted on the equity of the allocation of older adult resources from various aspects. First, studies have focused on specific provinces or municipalities, such as Hebei, Jiangsu, Yunnan, and Beijing (9–14), the capital cities of the provinces (15), older adult organizations (16), and older adult organizations in Chinese communities or rural areas (14, 17). Second, in terms of research methodology, various analytical methods, such as the Lorenz curve, Gini coefficient, and Thiel index (9, 12, 14, 15, 18, 19), concentration index (9, 10, 15), multiple spatial analysis methods (11), the health resource density index, entropy method and coupled coordinated evaluation model (16), analysis of the degree of agglomeration, and the DEA model (13) have been used. Third, the research indicators have been based on the number of older adult institutions (9, 10, 12–19), health technicians (9, 10, 12, 13, 19), employees (17, 18, 20), beds (11–15, 17–19), number of persons and days of service (16), the areas of older adult institutions (12, 16, 17, 20), percentage of registered nurses, expenditure on social older adult insurance, expenditure on social health insurance (20), fixed assets of older adult organizations, number of social workers and assistants (17).

In summary, researchers in recent years have mostly analyzed the equity of older adult resource allocation with a specific region, with few studies that have examined the overall situation for the 31 provinces (autonomous regions and municipalities) in mainland China, as well as for the East and the West. From the methodological aspect, different researchers have used different methods for analyzing the equity of older adult resource allocation, with different focuses, including the Lorenz curve and the Gini coefficient, which can reflect the degree of inequality in a region as a whole, express the results graphically and mathematically, and perform intuitively, but it is not possible to differentiate whether the inequality is caused by inter-region or intra-region differences (21). The Thiel index can determine whether inequity in a region is caused by inter-or intra-regional differences, but cannot consider the influence of geographical factors on the equity of health resource allocation (21). Agglomeration analysis can simultaneously consider the effects of population distribution and geographical size on the equity of health resource allocation and can dissect and digitally express regional inequities, allowing easy comparison (21). Therefore, this study selected a combination of methods including the Lorenz curve, the Gini coefficient, and agglomeration analysis. From research indicator aspect, to provide a more in-depth analysis of the situation of professional and technical personnel and in addition to the commonly used indicators of the number of older adult institutions and the number of beds, this study added two indicators, namely, the number of employees and the number of professional personnel.

The World Health Organization has proposed that the fairness of health resource allocation should meet the two important conditions of availability and accessibility. Availability, i.e., quantitative fulfillment, is necessary for the allocation of older adult resources according to the size of the older adult population, while accessibility indicates being geographically accessible, which is necessary for the allocation of older adult resources according to the geographical area; this is especially important in China’s western region, which has a large area and a small population (22). Therefore, the present study evaluated the equity of older adult resource allocation according to the two dimensions of geographical area and the size of the older adult population to ensure the objectivity and comprehensiveness of the fairness evaluation, aiming to provide a reference for the state as well as the government’s rational planning of older adult resources.

## 2 Methods

### 2.1 Sources

This study used data from 31 provinces, autonomous regions, and municipalities (excluding Taiwan, Hong Kong Special Administrative Region, and Macao Special Administrative Region) for analysis. We divided the eastern region, middle region, and western region according to the China Health Statistics Yearbook. The eastern region includes Beijing, Tianjin, Hebei, Liaoning, Shanghai, Jiangsu, Zhejiang, Fujian, Shandong, Guangdong, and Hainan (11 regions). The middle region includes Shanxi, Jilin, Heilongjiang, Anhui, Jiangxi, Henan, Hubei, and Hunan (8 regions). The western region includes Inner Mongolia, Chongqing, Guangxi, Sichuan, Guizhou, Yunnan, Tibet, Shaanxi, Gansu, Qinghai, Ningxia, and Xinjiang (12 regions).

The data sources included: data on the number of older adults over the age of 65 years old were obtained from the China Statistical Yearbook 2021 (23); data on geographical area were derived from the Red and Black Database (24); the older adult resources in this study refer to four indicators, namely, the number of older adult institutions, the number of employees, the number of professionals, and the number of beds in the older adult institution, which were derived from the China Civil Affairs Statistical Yearbook 2021 (25), in which the number of older adult institutions includes the sum of the data registered by the market supervision department, the establishment department, and the civil affairs department, as well as those registered by one institution with multiple brands; the number of employees refers to the total number of staff of older adult care institutions; the number of professional refers to the number of staff specializing in professional and technical skills, collected according to the nature of the staff; and the number of older adult care institution beds refers to the sum of the number of beds allocated to the institution. The number of beds refers to the sum of the number of beds of older adult institution (25).

### 2.2 Research methodology

Agglomeration analysis is derived from the field of industrial economics and has been increasingly used in health human resource allocation studies in recent years. The Health Work-Force Agglomeration Degree (HWAD) is defined as the amount of aggregated resources per 1% of the area or population of a region relative to the amount of aggregated resources per 1% of the area or population of the region at the previous level (21). We used agglomeration analysis to measure the degree of agglomeration of older adult resources in a particular region, as well as the differences among different groups. The agglomeration analysis of older adult resources was carried out in two dimensions based on the geographical area and the older adult population.

The formula used for the determination of the degree of agglomeration based on geographical area for older adult resources was HRAD*i* = (HR*_i_*/A*_i_*)/(HR*_n_*/A*_n_*), in which HR_i_ represents the number of older adult resources owned in an area i, A*_i_* is the land area of an area i, HR*_n_* is the total number of older adult resources in China, and A_n_ is the total land area in China (26). The formula for calculating the degree of agglomeration based on the older adult population for older adult resources was HRAD*_i_*/PAD*_i_* = (HR*_i_*/P*_i_*)/(HR*_n_*/P*_n_*), in which PAD*_i_* represents the degree of agglomeration of the older adult population in an area i. HR*_i_* and HR*_n_* have the same meaning as above. P*_i_* is the size of the older adult population in an area i, and P*_n_* represents the overall population of older adults in China (26). See Table 1 for the evaluation criteria (26).

**Table 1:** Evaluation criteria for agglomeration degree.

Agglomeration degree	HRAD*_i_*	HRAD*_i_*/PAD*_i_*
>1	Over-allocation by geographical area	Over-allocation by older adult population
=1	Absolute equity in allocation by geographical area	Absolute equity in allocation by older adult population
<1	Relative under-allocation by geographical area	Relative under-allocation by older adult population

The Gini coefficient is a measure of the equity of income distribution defined by the American economist Hirschman, which was first used to measure the equity of income distribution in the field of economics (27), and now the Gini coefficient has been widely used in the study of the equity of health resource allocation (28). The formula for calculating the Gini coefficient was *G* = 1-sigma (*X_i_-X_i -1_)(Y_i_ + Y_i -1_*), where X*_i_* is the cumulative percentage of the older adult population and geographical area of group i, and Y*_i_* is the cumulative percentage of older adult resources in group i (29). A Gini coefficient below 0.2 indicates absolute equity (very good state), while 0.2–0.3 indicates equity (good state), 0.3–0.4 indicates basic equity (normal state), 0.4–0.5 indicates inequity (alert status), and values greater than 0.5 indicate a high level of inequity (dangerous state) (29).

The Lorenz curve is a graphical representation of income inequality or wealth inequality proposed by the American economist Max Lorenz in 1905. The Lorenz curve is also widely used in the healthcare field and is often used to analyze the equity of health resources. The Lorenz curves, drawn according to the geographical area or the configuration of the older adult population, first rank the number of resources for the older adults per square kilometer or per 1,000 older adults in each region from smallest to largest, and then use the cumulative percentage of the older adult population or geographical area of each region as the horizontal coordinate, with the cumulative percentage of the resources for older adults as the vertical coordinate, and connect the points with smooth curves, where the diagonal lines represent the lines of absolute fairness (30). The more curved the Lorenz curve is, the more unequal the distribution of resources is; the flatter the Lorenz curve is, the more equal the distribution of resources is. According to the distance between the resulting curve and the absolute equity line, it can be seen that the smaller the distance, the more equitable the allocation of health resources, and vice versa, the more inequitable.

## 3 Results

### 3.1 Basic situation

#### 3.1.1 Allocation of older adult resources

From the analysis of the total older adult resources allocation in 31 provinces (autonomous regions and municipalities) in mainland China, as of 2020, there were overall 38,158 older adult institutions, 518,185 employees of older adult institutions, 378,897 professionals, and a total of 4,882,366 beds. Henan had the largest number of older adult institutions, namely, 3,244, while the numbers of employees and professionals, and the number of beds were highest in Jiangsu, being 46,882, 37,418, and 442,975, respectively. Analyzing the allocation of older adult resources per 1,000 older adults in 31 provinces (autonomous regions and municipalities) in mainland China, the number of older adult institutions per 1,000 older adults was 0.20, the number of employees per 1,000 older adults was 2.72, the number of professionals per 1,000 older adults was 1.99, and the number of beds per 1,000 older adults was 25.61. Among them, the number of older adult institutions per 1,000 older adults was highest in Jilin, with 0.4, the number of employees per 1,000 older adults was highest in Shanghai, at 0.40, while the number of beds per 1,000 older adults was lowest. Shanghai has the highest number of employees per 1,000 older adults population at 7.21, Beijing had the highest number of professionals per 1,000 older adults at 5.88, and Anhui had the highest number of beds per 1,000 older adults at 39.40 (see Table 2 for details).

**Table 2:** Distribution of older adult resources in 31 provinces (autonomous regions and municipalities) in China in 2020.

Area	Province (autonomous regions and municipalities)	older adult resources				Older adult resources per 1,000 older adult population			
		Older adult institution	Employee	Professional	Bed	Older adult institution	Employee	Professional	Bed
China		38,158	518,185	378,897	4,882,366	0.20	2.72	1.99	25.61
Eastern region	Beijing	584	20,283	17,127	112,848	0.20	6.97	5.88	38.75
	Tianjin	399	7,401	5,895	63,235	0.20	3.62	2.88	30.91
	Hebei	1726	29,535	23,494	231,981	0.17	2.84	2.26	22.33
	Liaoning	2035	19,241	12,953	178,035	0.27	2.59	1.75	24.00
	Shanghai	669	29,193	22,550	139,355	0.17	7.21	5.57	34.42
	Jiangsu	2,470	46,882	37,418	442,975	0.18	3.42	2.73	32.27
	Zhejiang	1752	26,607	19,896	335,694	0.20	3.11	2.32	39.19
	Fujian	640	8,759	6,304	84,312	0.14	1.90	1.37	18.29
	Shandong	2,190	38,359	30,636	358,829	0.14	2.50	1.99	23.36
	Guangdong	1891	32,041	24,906	251,597	0.17	2.96	2.30	23.27
	Hainan	48	1,136	850	9,002	0.05	1.08	0.81	8.56
Western region	Inner Mongolia	677	8,185	5,874	77,560	0.22	2.61	1.87	24.71
	Chongqing	927	12,083	8,805	102,560	0.17	2.21	1.61	18.74
	Guangxi	567	12,883	10,180	91,148	0.09	2.11	1.66	14.91
	Sichuan	2,541	21,202	12,005	297,638	0.18	1.50	0.85	21.01
	Guizhou	988	6,731	3,592	84,344	0.22	1.51	0.81	18.93
	Yunnan	880	7,667	5,891	89,142	0.17	1.51	1.16	17.57
	Tibet	23	433	349	4,160	0.11	2.09	1.69	20.10
	Shaanxi	735	12,276	9,179	105,009	0.14	2.33	1.74	19.94
	Gansu	268	3,301	2,255	29,049	0.09	1.05	0.72	9.23
	Qinghai	64	702	474	6,734	0.12	1.37	0.92	13.10
	Ningxia	112	1792	1,344	20,033	0.16	2.59	1.94	28.91
	Xinjiang	374	5,688	4,682	49,562	0.19	2.84	2.33	24.71
Middle region	Shanxi	665	8,807	5,793	72,262	0.15	1.96	1.29	16.04
	Jilin	1,498	17,686	11,544	136,103	0.40	4.71	3.07	36.22
	Heilongjiang	1709	16,584	12,430	165,563	0.34	3.33	2.50	33.29
	Anhui	2,452	24,169	15,725	360,892	0.27	2.64	1.72	39.40
	Jiangxi	1808	16,476	10,987	166,742	0.34	3.07	2.05	31.04
	Henan	3,244	34,404	23,959	310,535	0.24	2.57	1.79	23.17
	Hubei	1841	25,095	15,436	280,387	0.22	2.98	1.83	33.28
	Hunan	2,381	22,584	16,364	225,080	0.24	2.29	1.66	22.87

For sources, see Ministry of Civil Affairs of the People's Republic of China 25.

#### 3.1.2 Analysis of age and education level of older adult resources

Analysis of the ages of employees in older adult institutions in the 31 provinces (autonomous regions and municipalities) in mainland China showed that most employees were aged 46–55 years old, accounting for 34.82%, followed by 36–45 years old, accounting for 29.30%, of which, there were more employees aged 36–45 years old in Qinghai, accounting for 47.15%, while employees ≥56 years old were more common in Shanghai, accounting for 26.85%. Analysis of the education level of the employees of older adult institutions in 31 provinces (autonomous regions and municipalities) in mainland China indicated that the proportion of university graduates was 15.45% and the proportion of undergraduates and above was 8.25%, with the highest proportion of undergraduates and above found in the Ningxia, with a proportion of 16.29%. The highest proportion of university specialties was observed in Guangxi, with a proportion of 25.46%, and the lowest proportion of undergraduates and above was in Jilin, with a proportion of 2.8%, and the proportion of university specialties the lowest in Jilin, with a proportion of 4.5% (see Table 3 for details).

**Table 3:** Age and education level of employees in 31 provinces (autonomous regions and municipalities) in China.

	Province (autonomous regions and municipalities)	Total	Age of employee N(%)				Education level N(%)	
			≤35 years old	36–45 years old	46–55 years old	≥56 years old	Junior college	Bachelor degree or above
China		518,185	106,097 (20.47)	151,816 (29.30)	180,426 (34.82)	79,846 (15.41)	80,071 (15.45)	42,742 (8.25)
Eastern region	Beijing	20,283	3,814 (18.80)	4,022 (19.83)	8,860 (43.68)	3,587 (17.68)	3,017 (14.87)	1825 (9.00)
	Tianjin	7,401	1,344 (18.16)	2,446 (33.05)	2,448 (33.08)	1,163 (15.71)	960 (12.97)	824 (11.13)
	Hebei	29,535	6,177 (20.91)	8,849 (29.96)	9,978 (33.78)	4,531 (15.34)	4,076 (13.80)	1828 (6.19)
	Liaoning	19,241	3,534 (18.37)	5,748 (29.87)	6,854 (35.62)	3,105 (16.14)	2,667 (13.86)	1,230 (6.39)
	Shanghai	29,193	2,824 (9.67)	6,129 (20.99)	12,402 (42.48)	7,838 (26.85)	3,396 (11.63)	2006 (6.87)
	Jiangsu	46,882	11,756 (25.08)	12,697 (27.08)	15,204 (32.43)	7,225 (15.41)	8,513 (18.16)	5,060 (10.79)
	Zhejiang	26,607	4,888 (18.37)	7,466 (28.06)	9,146 (34.37)	5,107 (19.19)	3,231 (12.14)	1795 (6.75)
	Fujian	8,759	1828 (20.87)	2,293 (26.18)	2,829 (32.30)	1809 (20.65)	1,330 (15.18)	835 (9.53)
	Shandong	38,359	9,668 (25.20)	11,989 (31.25)	11,627 (30.31)	5,075 (13.23)	8,184 (21.34)	4,744 (12.37)
	Guangdong	32,041	6,504 (20.30)	8,894 (27.76)	12,153 (37.93)	4,490 (14.01)	4,618 (14.41)	3,386 (10.57)
	Hainan	1,136	356 (31.34)	315 (27.73)	397 (34.95)	68 (5.99)	159 (14.00)	94 (8.27)
Western region	Inner Mongolia	8,185	1,542 (18.84)	2,663 (32.54)	2,959 (36.15)	1,021 (12.47)	1,453 (17.75)	740 (9.04)
	Chongqing	12,083	2,142 (17.73)	2,748 (22.74)	4,667 (38.62)	2,526 (20.91)	1847 (15.29)	806 (6.67)
	Guangxi	12,883	3,754 (29.14)	3,941 (30.59)	3,661 (28.42)	1,527 (11.85)	3,280 (25.46)	1832 (14.22)
	Sichuan	21,202	4,838 (22.82)	6,892 (32.51)	6,609 (31.17)	2,863 (13.50)	3,879 (18.30)	1,517 (7.15)
	Guizhou	6,731	2031 (30.17)	2,317 (34.42)	1827 (27.14)	556 (8.26)	1,552 (23.06)	745 (11.07)
	Yunnan	7,667	2,352 (30.68)	2,688 (35.06)	2,142 (27.94)	485 (6.33)	1,427 (18.61)	954 (12.44)
	Tibet	433	295 (68.13)	122 (28.18)	14 (3.23)	2 (0.46)	39 (9.01)	31 (7.16)
	Shaanxi	12,276	3,779 (30.78)	3,771 (30.72)	3,345 (27.25)	1,381 (11.25)	2,709 (22.07)	1,329 (10.83)
	Gansu	3,301	1,013 (30.69)	1,358 (41.14)	763 (23.11)	167 (5.06)	783 (23.72)	359 (10.88)
	Qinghai	702	163 (23.22)	331 (47.15)	197 (28.06)	11 (1.57)	95 (13.53)	71 (10.11)
	Ningxia	1792	475 (26.51)	711 (39.68)	483 (26.95)	123 (6.86)	338 (18.86)	292 (16.29)
	Xinjiang	5,688	1,617 (28.43)	1992 (35.02)	1702 (29.92)	377 (6.63)	1,016 (17.86)	461 (8.10)
Middle region	Shanxi	8,807	2,238 (25.41)	2,768 (31.43)	2,645 (30.03)	1,156 (13.13)	1,456 (16.53)	653 (7.41)
	Jilin	17,686	1,573 (8.89)	4,175 (23.61)	10,809 (61.12)	1,129 (6.38)	795 (4.50)	496 (2.80)
	Heilongjiang	16,584	3,414 (20.59)	5,980 (36.06)	5,418 (32.67)	1772 (10.68)	2040 (12.30)	976 (5.89)
	Anhui	24,169	4,467 (18.48)	7,347 (30.40)	8,410 (34.80)	3,945 (16.32)	3,470 (14.36)	1,441 (5.96)
	Jiangxi	16,476	2,498 (15.16)	3,943 (23.93)	5,845 (35.48)	4,190 (25.43)	2030 (12.32)	708 (4.30)
	Henan	34,404	6,253 (18.18)	9,611 (27.94)	12,058 (35.05)	6,482 (18.84)	4,061 (11.80)	1993 (5.79)
	Hubei	25,095	4,128 (16.45)	10,127 (40.35)	7,411 (29.53)	3,429 (13.66)	3,343 (13.32)	1,374 (5.48)
	Hunan	22,584	4,832 (21.40)	7,483 (33.13)	7,563 (33.49)	2,706 (11.98)	4,307 (19.07)	2,337 (10.35)

For sources, see Ministry of Civil Affairs of the People's Republic of China 25.

### 3.2 Agglomeration analysis of older adult resources allocation

The older adult resource allocation in the 31 provinces (autonomous regions and municipalities) in mainland China was analyzed in terms of the degree of agglomeration according to two dimensions, namely, the geographical area and the size of the older adult population.

#### 3.2.1 Agglomeration analysis of older adult resource allocation by geographical area

According to the analysis of geographical area allocation, the degrees of agglomeration of older adult institutions, employees, professionals, and beds in Tibet, Qinghai, Xinjiang, Inner Mongolia, and Gansu were lower than 0.20, while the degree of agglomeration of older adult institutions in Hainan and Ningxia was lower than 0.45, while that of older adult institutions in Jiangsu, Tianjin, Beijing, and Shanghai was over 5, and that of employees, professionals, and beds was higher than 8. The results of the analysis of the four indicators of agglomeration showed that in terms of the agglomeration of older adult institutions, Shanghai (26.803) was the highest, Tibet (0.005) was the lowest, and Shanxi (1.071) was the closest to 1. In terms of the agglomeration of employees, Shanghai (86.128) was the highest, Tibet (0.007) was the lowest, and Guangxi (1.008) was the closest to 1. In terms of the agglomeration of professional, Shanghai (90.986) was the highest and Tibet (0.007) was the lowest, while Guangxi (1.089) was closest to 1, and in terms of the agglomeration of beds, Shanghai (43.636) was the highest, Tibet (0.007) was the lowest, and Shaanxi (1.008) was closest to 1 (see Table 4 for details).

**Table 4:** Agglomeration analysis of older adult resource allocation by geographical area.

Area	Province (autonomous regions and municipalities)	Geographical area (10.000 km^2^)	Older adult institution	Employee	Professional	Bed
Eastern region	Beijing	1.64	8.988	22.988	26.546	13.574
	Tianjin	1.2	8.393	11.463	12.487	10.395
	Hebei	18.88	2.308	2.908	3.163	2.424
	Liaoning	14.86	3.457	2.407	2.216	2.363
	Shanghai	0.63	26.803	86.128	90.986	43.636
	Jiangsu	10.72	5.816	8.129	8.873	8.152
	Zhejiang	10.35	4.273	4.778	4.886	6.398
	Fujian	12.14	1.331	1.341	1.320	1.370
	Shandong	15.79	3.501	4.515	4.932	4.483
	Guangdong	17.97	2.656	3.314	3.523	2.762
	Hainan	3.54	0.342	0.596	0.610	0.502
Western region	Inner Mongolia	118.3	0.144	0.129	0.126	0.129
	Chongqing	8.24	2.840	2.726	2.716	2.455
	Guangxi	23.76	0.602	1.008	1.089	0.757
	Sichuan	48.6	1.320	0.811	0.628	1.208
	Guizhou	17.61	1.416	0.710	0.518	0.945
	Yunnan	39.41	0.564	0.362	0.380	0.446
	Tibet	122.84	0.005	0.007	0.007	0.007
	Shaanxi	20.56	0.902	1.110	1.135	1.008
	Gansu	42.58	0.159	0.144	0.135	0.135
	Qinghai	72	0.022	0.018	0.017	0.018
	Ningxia	6.64	0.426	0.502	0.515	0.595
	Xinjiang	166	0.057	0.064	0.072	0.059
Middle region	Shanxi	15.67	1.071	1.045	0.940	0.910
	Jilin	18.74	2.018	1.754	1.566	1.433
	Heilongjiang	47.3	0.912	0.652	0.668	0.690
	Anhui	14.01	4.418	3.206	2.853	5.082
	Jiangxi	16.69	2.734	1.835	1.673	1.971
	Henan	16.7	4.903	3.829	3.647	3.668
	Hubei	18.59	2.500	2.509	2.111	2.975
	Hunan	21.18	2.838	1.982	1.964	2.096

For sources, see Red and Black Database (24) and Ministry of Civil Affairs of the People's Republic of China (25).

#### 3.2.2 Agglomeration analysis of older adult resources according to the size of the older adult population

In terms of the size of the older adult population, the degree of agglomeration of the allocation of older adult institutions in Hainan was lower than 0.25, while that of the allocation of employees in Gansu and Hainan was below 0.4, the agglomeration degree of the allocation of professionals in Gansu, Guizhou, and Hainan was lower than 0.41, and that of the allocation of beds in Hainan and Gansu was lower than 0.37. The degree of agglomeration of the allocation of older adult institutions in Heilongjiang and Jilin was higher than 1.71, that of the allocation of employees in Jilin, Beijing, and Shanghai was higher than 1.73, while Shanghai and Beijing’s agglomeration of professional allocation was higher than 2.8, and Beijing, Zhejiang, and Anhui’s agglomeration of bed allocation was higher than 1.5. Analysis of the four indicators of agglomeration showed that the agglomeration of the older adult institution allocation in Jilin (1.992) was the highest, while Hainan (0.228) was the lowest, and Beijing (1.002) was the closest to 1. The agglomeration of employee allocation was highest in Shanghai (2.652), and lowest in Gansu (0.386), while Anhui (0.971) was the closest to 1. The agglomeration of professional allocation was highest in Beijing (2.959), lowest in Gansu (0.360), and Shandong (1.003) was the closest to 1. The agglomeration of bed allocation was highest in Anhui (1.538), lowest in Hainan (0.334), and the closest to 1 in Xinjiang and Inner Mongolia (0.965) (see Table 5 for details).

**Table 5:** Agglomeration analysis of older adult resource allocation based on the size of the older adult population.

Area	Province (autonomous regions and municipalities)	Population aged 65 and above	PAD	HRAD*_i_*/PAD*_i_*			
				Older adult institution	Employee	Professional	Bed
Eastern region	Beijing	2,912,060	8.971	1.002	2.562	2.959	1.513
	Tianjin	2,045,692	8.613	0.974	1.331	1.450	1.207
	Hebei	10,387,937	2.780	0.830	1.046	1.138	0.872
	Liaoning	7,417,481	2.522	1.371	0.954	0.879	0.937
	Shanghai	4,049,012	32.471	0.825	2.652	2.802	1.344
	Jiangsu	13,726,531	6.469	0.899	1.257	1.372	1.260
	Zhejiang	8,566,349	4.182	1.022	1.143	1.169	1.530
	Fujian	4,609,999	1.919	0.694	0.699	0.688	0.714
	Shandong	15,364,078	4.916	0.712	0.919	1.003	0.912
	Guangdong	10,813,000	3.040	0.874	1.090	1.159	0.909
	Hainan	1,051,500	1.501	0.228	0.397	0.407	0.334
Western region	Inner Mongolia	3,138,918	0.134	1.078	0.959	0.942	0.965
	Chongqing	5,473,605	3.356	0.846	0.812	0.809	0.732
	Guangxi	6,114,117	1.300	0.463	0.775	0.838	0.582
	Sichuan	14,167,600	1.473	0.896	0.551	0.426	0.820
	Guizhou	4,456,455	1.279	1.108	0.556	0.406	0.739
	Yunnan	5,073,259	0.650	0.867	0.556	0.584	0.686
	Tibet	206,963	0.009	0.555	0.770	0.848	0.785
	Shaanxi	5,266,597	1.294	0.697	0.858	0.877	0.779
	Gansu	3,147,817	0.373	0.425	0.386	0.360	0.360
	Qinghai	514,093	0.036	0.622	0.502	0.464	0.511
	Ningxia	692,824	0.527	0.808	0.952	0.976	1.129
	Xinjiang	2,005,885	0.061	0.932	1.043	1.174	0.965
Middle region	Shanxi	4,504,674	1.452	0.738	0.719	0.647	0.626
	Jilin	3,757,224	1.013	1.992	1.732	1.546	1.414
	Heilongjiang	4,972,868	0.531	1.717	1.227	1.258	1.300
	Anhui	9,159,411	3.303	1.337	0.971	0.864	1.538
	Jiangxi	5,371,021	1.626	1.682	1.129	1.029	1.212
	Henan	13,401,904	4.054	1.209	0.944	0.899	0.905
	Hubei	8,424,339	2.290	1.092	1.096	0.922	1.300
	Hunan	9,842,067	2.348	1.209	0.844	0.837	0.893

For sources, see National Bureau of Statistics of China (23), Red and Black Database (24) and Ministry of Civil Affairs of the People's Republic of China (25).

### 3.3 Gini coefficients and Lorenz curves of older adult resource allocation

#### 3.3.1 Gini coefficient of older adult resource allocation in 31 provinces (autonomous regions and municipalities) of mainland China

**Figure 1:**
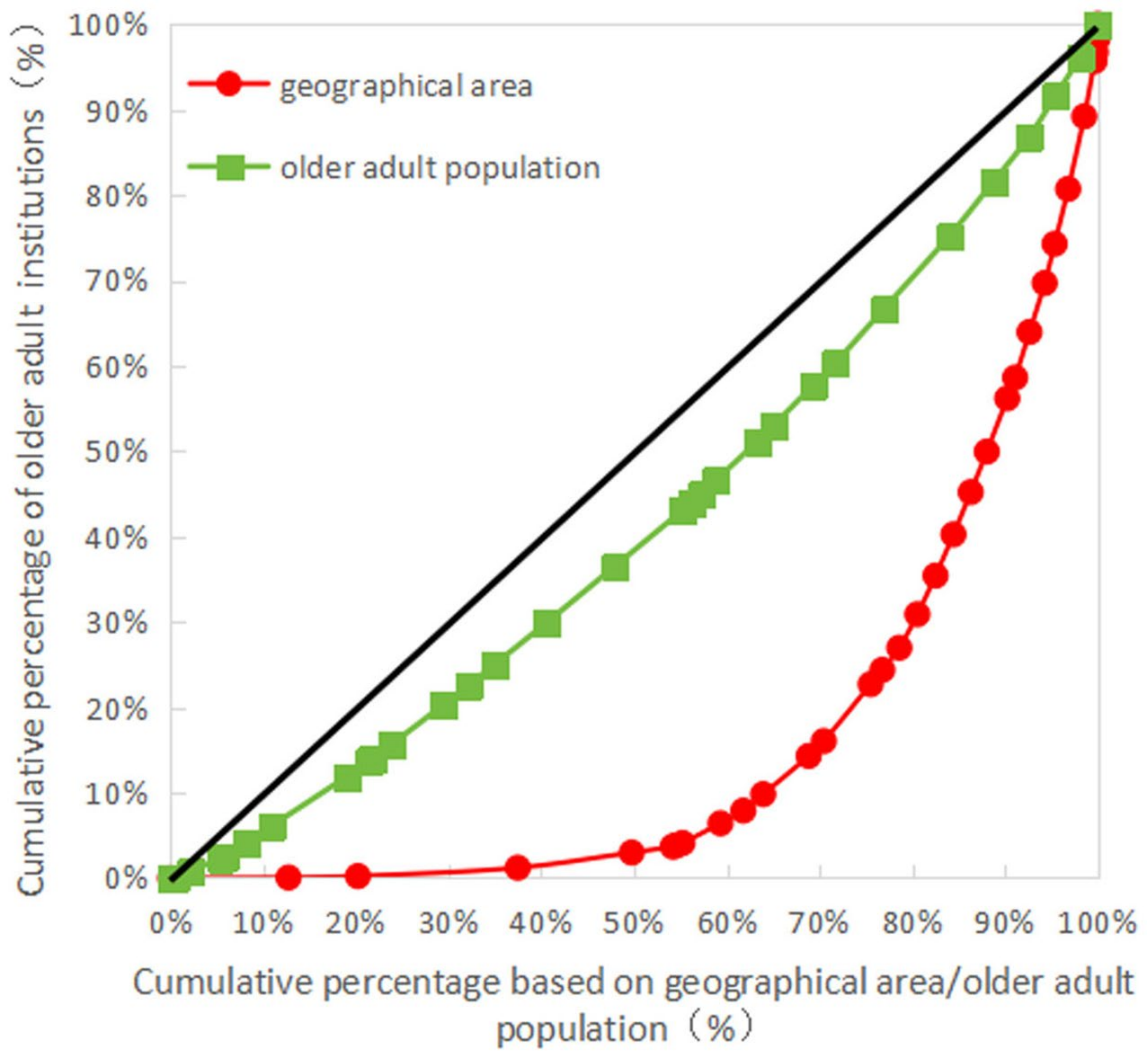
Lorenz for allocation of older adult institutions in China.

**Figure 2:**
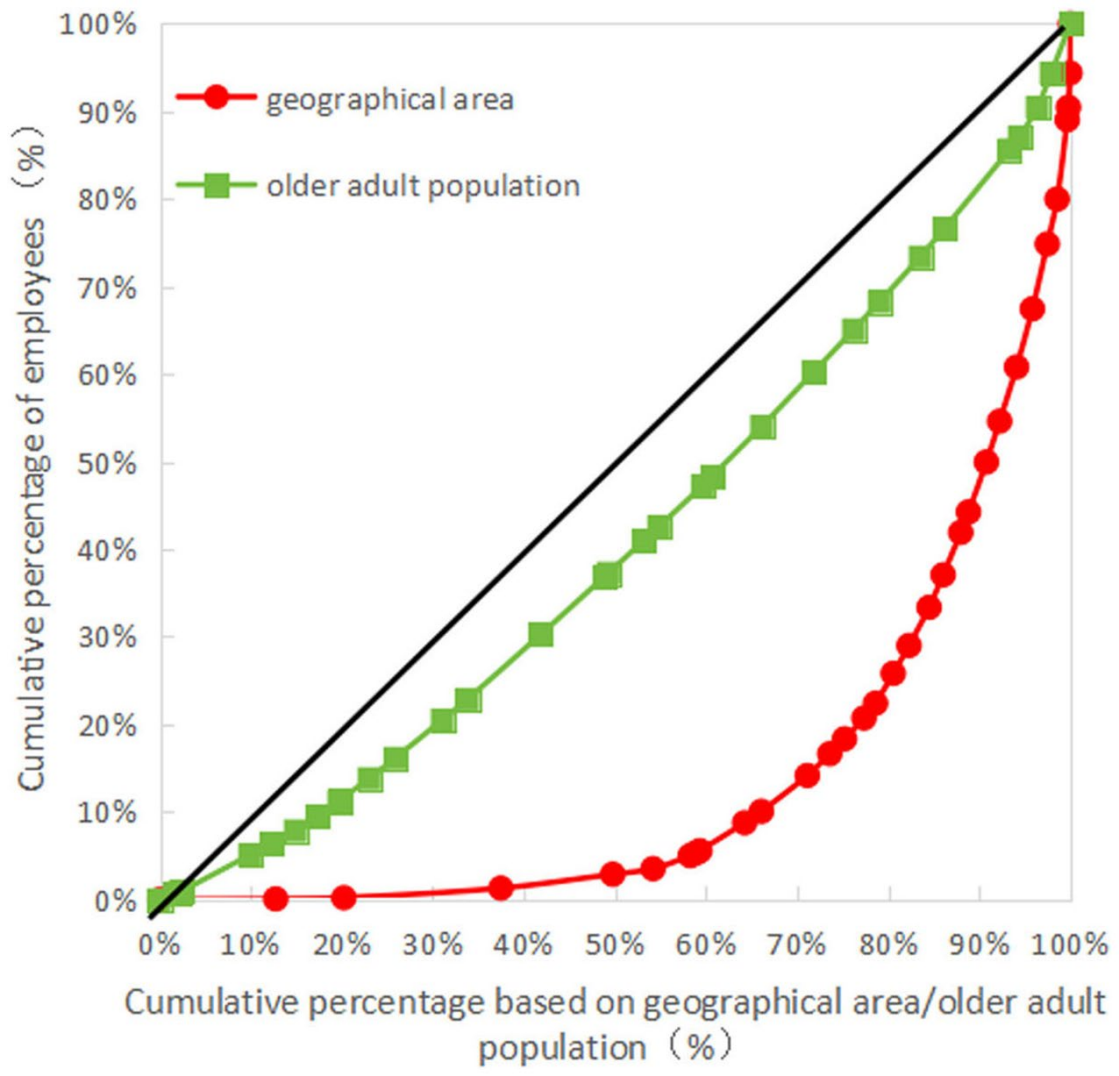
Lorenz curve for allocation of employees in older adult institutions in China.

**Figure 3:**
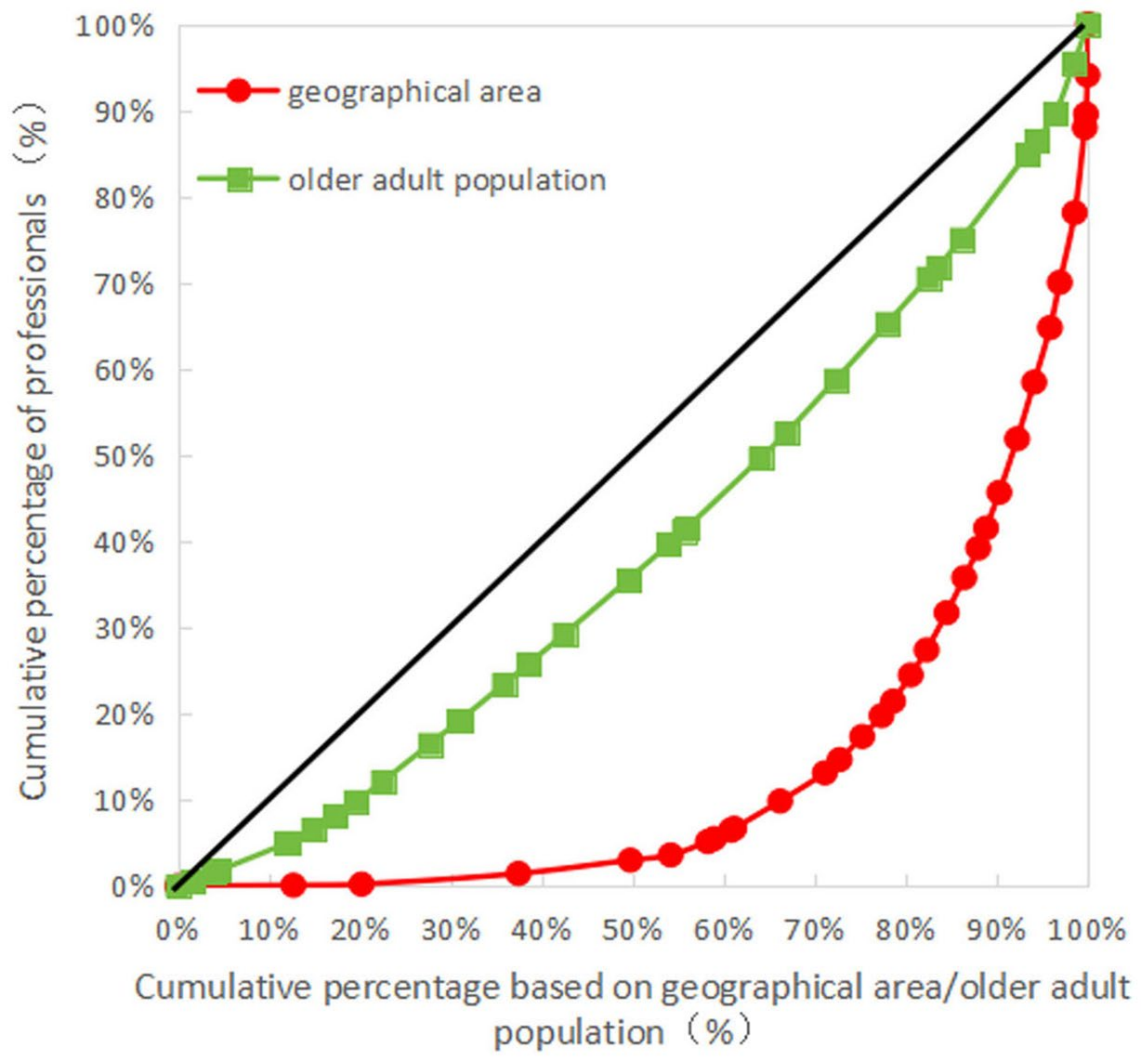
Lorenz curve for allocation of professionals in older adult institutions in China.

**Figure 4:**
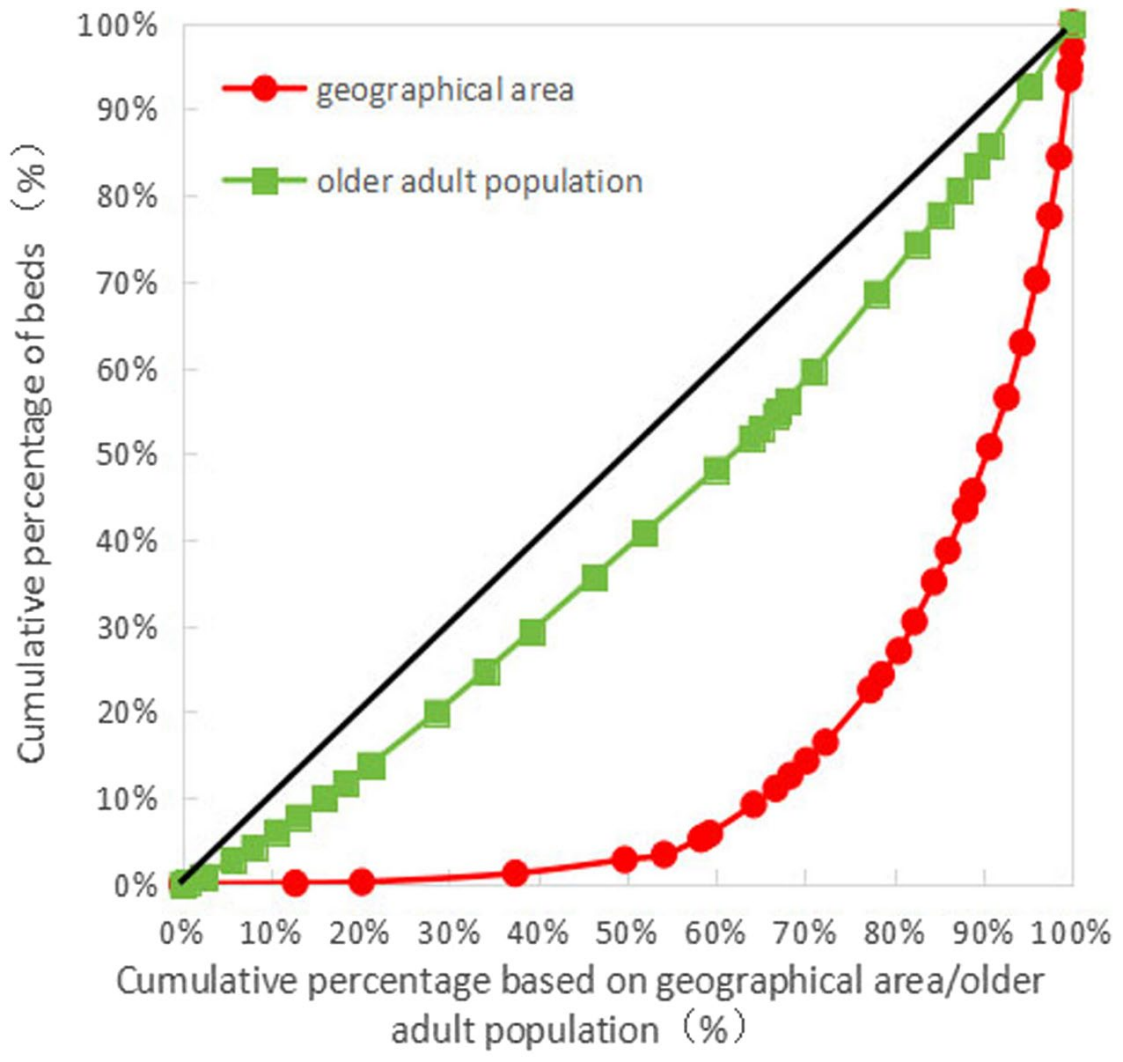
Lorenz curve for allocation of beds in older adult institutions in China.

The Gini coefficient of older adult resources in terms of geographical area allocation showed that the Gini coefficients of the number of older adult institutions, the number of employees, the number of professionals, and the number of beds were all more than 0.65, indicating that the allocation of resources in each province (autonomous regions and municipalities) is very unfair and is in a dangerous state. The Gini coefficient of older adult resources based on the older adult population showed that the Gini coefficient of the number of professionals was less than 0.3, while those of the number of older adult institutions, employees, and beds were all less than 0.2, indicative of fairness in the allocation of resources (see Table 6 for details). The Lorenz curves drawn based on the Gini coefficients are detailed in Figures 1–4.

**Table 6:** Gini coefficients for older adult resource allocation according to different dimensions.

Resource category	Gini coefficient (by geographical area)	Gini coefficient (by older adult population)
Older adult institution	0.685	0.170
Employee	0.727	0.186
Professional	0.736	0.215
Bed	0.715	0.159

#### 3.3.2 Gini coefficients of older adult care resource allocation in different regions of mainland China

Analysis of data from the eastern, middle, and western regions of China showed that the Gini coefficients of the older adult resources based on the allocation of geographical area in terms of the numbers of older adult institutions, employees, professionals, and beds were all greater than 0.65 in the western region, indicating that the allocation according to geographical area is very unfair in the western region, and is in a dangerous state, while the Gini coefficients of the older adult resources based on the allocation of geographical area were lower than 0.4 in the eastern and middle regions. This indicated that resource allocation in these regions was basically fair and in a normal state, and the equity of the number of older adult institutions in the eastern region was better than that in the middle region, although the equity in terms of the number of employees, professionals, and beds was lower than that in the middle region. The Gini coefficients of older adult resources based on the older adult population showed that the values in the eastern, middle, and western regions were all less than 0.2, indicating that the allocation of older adult resources according to the older adult population was absolutely fair in the eastern, middle, and western regions, and was in a very good state (see Table 7 for details).

**Table 7:** The regional distribution of Gini coefficients of older adult resources according to different dimensions.

Area	Gini coefficient (by geographical area)				Gini coefficient (by older adult population)			
	Older adult institution	Employee	Professional	Bed	Older adult institution	Employee	Professional	Bed
Eastern region	0.281	0.397	0.414	0.371	0.111	0.170	0.184	0.131
Western region	0.699	0.678	0.670	0.689	0.134	0.140	0.200	0.105
Middle region	0.313	0.312	0.302	0.361	0.121	0.108	0.102	0.137

#### 3.3.3 Lorenz curves of older adult resource allocation in 31 provinces (autonomous regions and municipalities) in mainland China

As can be seen in Figures 1–4, for the older adult resources configured by the older adult population, the equity of the configuration of the number of beds was the best, with a Gini coefficient of 0.159. In terms of older adult resources configured by geographical area, all the indices showed that the configuration was inequitable, among which the equity of the number of older adult institution configurations was better than the other indices, with a Gini coefficient of 0.685, which is still in a dangerous state (see Figures 1–4 for details).

## 4 Discussion

The allocation of older adult resources in 31 provinces (autonomous regions and municipalities) in mainland China has increased and to a certain extent, the needs of older adults have been met (16). Data analysis showed that China’s older adult resources, including older adult institutions, employees, professionals, and beds, have been increasing in recent years, with the four indicators reaching 38,158, 518,185, 378,897, and 4,882,366, respectively, by the end of 2020. The proportion of employees with an educational level of college or above in older adult institutions was found to be relatively low, at 23.7%, while the age structure of the employees was essentially aging. Among them, those over 45 years old accounted for 50.23%. The number of institutions, employees, professionals, and beds per 1,000 older adults reached 0.2, 2.72, 1.99, and 25.61, respectively, with the maximum values of the above indicators being 0.4, 7.21, 5.88, and 39.4, and the minimum values 0.005, 1.05, 0.72, and 8.56, respectively, and with great variability in different provinces (autonomous regions, municipalities).

Currently, China’s old adult care model is based on home-based care, supplemented by institutions, but with the aging of the population and the phenomenon of childlessness, the demand for older adult institutions will continue to increase. China has put forward a national strategy for actively coping with population aging during the “14th Five-Year Plan” period, which aims to improve the old adult service system and health support system, and to enhance the sense of accessibility, happiness and security of the older adult (6). *The”14th Five-Year Plan” for the Development of the National Aging Career and Senior Care Service System* proposes that by 2025, the number of social workers per 1,000 seniors will remain at more than one, and the number of beds in senior care institutions will reach more than 9 million; these policies and measures, as well as the target plan, provide an important reference for actively coping with the aging of the population (7).

The 31 provinces (autonomous regions and municipalities) in mainland China were found to have wide disparities in the equity of older adult resource allocation, with both over-and under-allocation of older adult resources, and the disparity in allocation in terms of geographical area was particularly obvious. The results of the allocation of older adult resources in terms of geographical area showed that there was a clear shortage of older adult resources in Tibet, Qinghai, Xinjiang, Inner Mongolia, Gansu, Hainan, and Ningxia, which, apart from Hainan, are distributed in the western region, while there is a clear over-allocation of older adult resources in Jiangsu, Tianjin, Beijing, and Shanghai, most of which are located in the eastern region. These results are consistent with published findings (16, 18, 19). The results of the allocation of older adult resources according to the older adult population showed that Hainan, Gansu, Guizhou, Guangxi, and other regions were slightly under-allocated in terms of older adult resources; these, apart from Hainan, are located mainly in the western region, while Heilongjiang, Jilin, Beijing, Shanghai, Zhejiang, Anhui, and other areas, situated mainly in the eastern and middle regions, were slightly over-allocated; these results are consistent with published findings (16, 18, 19). In the western regions, such as Tibet, Qinghai, and Xinjiang, older adult resources were insufficiently allocated according to geographical areas, due to the large geographical areas and sparse populations of these regions. The problem of low accessibility of older adult resources is highlighted, which is consistent with the conclusions of previous studies (10). From an economic perspective, the centralized allocation of older adult resources in economically developed provinces (autonomous regions and municipalities) is mainly due to the high demand for services from residents in those areas, as well as the regulation of market mechanisms, consistent with the findings of earlier research (10, 15). However, some researchers have found that underdeveloped cities require more consideration in the distribution of professionals for older adult resources (9). These different conclusions may be attributed to differences in the indicators used for the evaluation of older adult resources as well as the types of public and private resources.

The older adult resources in China were found to be more equitably allocated according to the older adult population, but less so according to geographical area, with large differences seen among the eastern, middle, and western regions. The Gini coefficient analysis found that the allocation of older adult resources according to the dimension of the geographical area was very unfair and in a dangerous state. Analysis of the older adult resource allocation according to the size of the older adult population was found to be fair and in a good state. Analysis of the Gini coefficient in the eastern, middle, and western regions revealed that analyzed according to the dimension of geographical area, the allocation in the western region was very unfair, with Gini coefficients above 0.6, for which the main reasons were the sparse population and large geographical area of the western region, while the allocation in the eastern and middle regions was essentially fair. These results are consistent with the findings of published studies (15). When analyzed in terms of the size of the older adult population, the overall allocation in the eastern, middle, and western regions showed fairness, except for the indicator of allocation according to the number of beds in which the western regions scored better than the middle and eastern regions, while the fairness in the allocation of the other indicators tended to be better in the eastern and middle regions. While these findings are consistent with the general trend of published research, they cannot be compared directly due to differences in the choice of indicators used by the various researchers (15, 18, 19).

It is suggested that firstly, the government should strengthen the professional teams of older adult care institutions, to form a reasonable structure in terms of age, education, and title of high-quality professional and technical personnel (31, 32). Secondly, for the western region where the allocation of old adult resources is insufficient, the government should give full play to the role of macro-control and comprehensively consider the geographical area and distribution of the older adult population in the different provinces (autonomous regions and municipalities), and target the allocation and planning of resources for older adults to ensure that residents in the western region are able to access resources (33, 34). Thirdly, for economically developed provinces (autonomous regions and municipalities) in the eastern and middle regions, social capital should be introduced to establish different types of older adult care institutions to meet the high demands for older adult care services, and at the same time, government funds should be invested more in the western regions, to realize a balanced allocation of older adult care resources in the east, middle, and western regions of China, as well as a multilevel development (35–37).

### 4.1 Limitations

First, this study evaluated the equity of older adult resource allocation based on the assumption of resource homogeneity and did not address differences in service quality and service capacity of different older adult institutions and professionals. Second, data on the older adult resources in this study were derived from data on older adult institutions provided by the China Civil Affairs Statistical Yearbook; however, the older adult resources in China include not only older adult institutions, but also other older adult resources such as home-based care of older adults, community-based older adult care, and older adult care in medical and healthcare institutions, which are not considered in this study due to the lack of data sources for the above information, and thus the conclusions reached may differ from the conclusions taking the allocation of all older adult resources into account. Third, since there is no specific evaluation standard for the Gini coefficient of older adult resource allocation, the study drew on the evaluation standard for income equity in economics, and the conclusion may thus be affected.

## Data availability statement

The original contributions presented in the study are included in the article/supplementary material, further inquiries can be directed to the corresponding author.

## Author contributions

QY: Methodology, Resources, Writing – original draft, Writing – review & editing. TZ: Writing – review & editing, Validation. LJ: Writing – review & editing. YJ: Writing – review & editing. YD: Writing – review & editing. LL: Writing – review & editing.

## References

[1] Chen W Guo YL. Negative population growth and population aging in China. Beijing Soc Sci. (2023) 8:101–12. doi: 10.13262/j.bjsshkxy.bjshkx.230810

[2] Zhang ZZ. Analysis on the balance and efficiency of old adult service resource allocation in China. Zhongnan University of Econ and Law (2022). doi: 10.27660/d.cnki.gzczu.2022.002551

[3] Xia CC Lin B. International experience in coping with population aging and implications for China's population policy. Soc Sci J. (2023) 5:148–57.

[4] The State Council of the People’s Republic of China. CPC central committee state council issues medium-long-term plan for the state to actively cope with population aging. (2019). Available at: https://www.gov.cn/xinwen/2019-11/21/content_5454347.htm (Accessed January 1, 2024).

[5] The State Council of the People’s Republic of China. Opinions of the central committee of the communist party of China and the state council on strengthening the work of the old adult in the New Era.(2021). Available at: https://www.gov.cn/zhengce/2021-11/24/content_5653181.htm?eqid=bb8541fd00009a9f000000036487d155 (Accessed January 1, 2024).

[6] The State Council of the People’s Republic of China. (2022). Circular of the state council on the issuance of the 14th five-year plan for the development of the national aging career and older adult service system. Available at: https://www.gov.cn/gongbao/content/2022/content_5678066.htm?eqid=cae4cd050009b1280000000264870a79 (Accessed January 1, 2024).

[7] The News Network of the Communist Party of China. (2022). Holding high the great banner of socialism with Chinese characteristics and striving in unity for the comprehensive construction of a modernized socialist country-report at the 20th National Congress of the Communist Party of China. Available at: http://cpc.people.com.cn/n1/2022/1026/c64094-32551700.html (Accessed January 1, 2024).

[8] Li HM. A review of research on the impact of population aging on residents' healthcare expenditure. Times Finance. (2023) 10:54–55+80. doi: 10.3969/j.issn.1672-8661(s).2023.10.020

[9] Niu XH. A study on the allocation of urban old adult care resources in Hebei based on spatial feature. Yanshan University. (2023). doi: 10.27440/d.cnki.gysdu.2023.001582

[10] Meng TJ Xue MT Bu ZH Li QY Peng XZ Xu GH. Current status and equity of geriatric nursing human resources in Jiangsu Province based on the concentration index. Chin J Gerontol. (2019) 13:285–90.

[11] Bi XY Li M. Equity vs. efficiency: a spatial analysis of residential aged care resources in Beijing. Chin J Sociol. (2020) 40:117–47. doi: 10.15992/j.cnki.31-1123/c.2020.03.005

[12] Wang MY Wang F Zeng DH Ding CF Zheng X Zhao YX. Equity analysis of Wuhan's institutional senior care resource allocation under the combined medical and nursing care model. China Health Stat. (2018) 35:462–463+465.

[13] Zhang X. Analysis of equity and efficiency of older adult resource allocation in Yunnan Province. China Collect Econ. (2023) 7:165–8. doi: 10.3969/j.issn.1008-1283.2023.7.zgjtjj202307047

[14] Zhang XD Song Q. Research on the equity of the allocation of county-level old adult service resources in Yunnan Province. China Coll Econ. (2022) 29:164–8. doi: 10.3969/j.issn.1008-1283.2022.29.zgjtjj202229055

[15] Li F Gao XD. On the equilibrium of the allocation of social older adult resources in China: a perspective based on the differences between provincial capital cities. Popul Soc. (2019) 35:48–56. doi: 10.14132/j.2095-7963.2019.05.004

[16] Bian JW Rao KQ. Spatio-temporal evolution of the coordinated development of China's older adult resources allocation and service utilization: based on the hierarchical analysis framework of institutions. Chin J Health Policy. (2022) 15:30–40. doi: 10.12201/bmr.202209.00014

[17] Li M Ao Y Peng P Bahmani H Han L Zhou Z. Resource allocation of rural institutional old adult care in China's new era: spatial-temporal differences and adaptation development. Public Health. (2023) 223:7–14. doi: 10.1016/j.puhe.2023.07.005, PMID: 37572563

[18] Sun SJ. Empirical analysis of the equity of older adult resources allocation in China under the background of population aging. J Chengdu Normal Coll. (2022) 38:107–12. doi: 10.3969/j.issn.2095-5642.2022.02.015

[19] Li CY Zhang XY. An empirical investigation on the equalization of resource allocation for community old adult services in China--based on the Terrell index measurement and decomposition analysis. Local Finance Res. (2023) 6:28–39.

[20] Peng R Huang J Deng X. Spatiotemporal evolution and influencing factors of the allocation of social care resources for the older adults in China. Int J Equity Health. (2023) 22:222. doi: 10.1186/s12939-023-02007-0 37853486 PMC10583468

[21] Zhu B Mao Y He RX Zhang N Ning W Liu JL. Reviewing the methodology of equity in health human resources allocation in China and its application. Chin Health Serv Manag. (2022) 39:10–6.

[22] Campbell J Buchan J Cometto G David B Dussault G Fogstad H. Human resources for health and universal health coverage: fostering equity and effective coverage. Bull World Health Organ. (2013) 91:853–63. doi: 10.2471/BLT.13.118729, PMID: 24347710 PMC3853950

[23] National Bureau of Statistics of China. China statistical yearbook 2021. (2021). Available at: https://www.stats.gov.cn/sj/ndsj/2021/indexch.htm (Accessed January 1, 2024).

[24] Red and Black Database. (2020). Red and black database: provincial jurisdictional areas. Available at: https://www.hongheiku.com/ (Accessed January 1, 2024).

[25] Ministry of Civil Affairs of the People's Republic of China. China civil affairs statistical yearbook 2021 (in Chinese). Beijing: China Society Press (2021).

[26] Yuan SW Wei FQ Liu WW Zhang Z Ma J. Methodology discussion of health resource allocation equity evaluation based on agglomeration degree. Chin Hosp Manag. (2015) 35:3–5.

[27] Han XY Chen P Zhang JH Chen Y He LP Meng Q. An empirical study of different algorithms of Gini coefficient in the study of equity of health resource allocation. China Health Statistics. (2021) 38:128–30. doi: 10.3969/j.issn.1002-3674.2021.01.033

[28] Chen AQ Xu AJ Xue CB. Equity analysis of health resource allocation in Jiangsu Province based on Gini coefficient and agglomeration. China Health Statistics. (2018) 35:527–9. doi: CNKI:SUN:ZGWT.0.2018-04-011

[29] Feng LF Xie YX Chen L. Analysis of fairness in nursing human resource allocation in Guangdong Province based on Gini coefficient and Theil index. Modern Hosp. (2021) 21:61–3. doi: 10.3969/j.issn.1671-332X.2021.01.018

[30] Zhang HY Miao YD Qu XY Wang LY Wang JZ Gu JQ. A study on the equity of resource allocation of general practitioners in China based on Lorenz curve and Gini coefficient. Chin Gen Pract Med. (2020) 23:409–13. doi: 10.12114/j.issn.1007-9572.2019.00.783

[31] Wang F Feng Y. Equity analysis of health resource allocation in Guizhou province based on agglomeration and rank sum ratio method. Modern Prevent Med. (2022) 49:2388–92. doi: 10.20043/j.cnki.MPM.202203266

[32] Sun J Wang QQ Wen QL. Evaluation of the current status of health resource allocation in Guangxi based on the rank-sum ratio method. China Health Statist. (2017) 34:488–489+491.

[33] Zhang L Wang WH Li T. Equity analysis of health human resource allocation in Shaanxi Province based on agglomeration. China Health Q Manag. (2022) 29:102–6. doi: 10.13912/j.cnki.chqm.2022.29.07.25

[34] Zhao LP Ye BZ Xing YH Zhang WD Wu J. Equity and spatial distribution of human resource allocation for maternal and child health in China from 2012 to 2020. Health Soft Science. (2023) 37 62–66+76. doi: 10.3969/j.issn.1003-2800.2023.10.013

[35] He LS Xiong WY Wang M Zhao S Fu X Ma WS. Analysis of the status quo and equity of human resource allocation in primary healthcare organizations in Tianjin from 2015 to 2020. Occupat Health. (2023) 39:1856–60. doi: 10.13329/j.cnki.zyyjk.2023.0315

[36] Zang H He C Wei RN Huang M Du XP. Analysis of rationality of health resource allocation in China based on agglomeration. Health Soft Sci. (2021) 35:59–64. doi: 10.3969/j.issn.1003-2800.2021.10.013

[37] Hu TY Pei YL Ma Y You Y Xie NQ. Research on the equity of nursing human resource allocation and demand forecasting in medical and healthcare organizations in the western region of China. Modern Prevent Med. (2023) 50:3367–72. doi: 10.20043/j.cnki.MPM.202305354

